# Endovascular management of giant visceral artery aneurysms

**DOI:** 10.1038/s41598-020-80150-2

**Published:** 2021-01-12

**Authors:** Marcello Andrea Tipaldi, Miltiadis Krokidis, Gianluigi Orgera, Matteo Pignatelli, Edoardo Ronconi, Florindo Laurino, Andrea Laghi, Michele Rossi

**Affiliations:** 1 Department of Radiology, Sant’ Andrea University Hospital La Sapienza, Rome, Italy; 2 Department of Surgical and Medical Sciences and Translational Medicine, School of Medicine and Psychology, “Sapienza”, University of Rome, Rome, Italy; 3 School of Medicine, National and Kapodistrian University of Athens, Areteion Hospital, 76, Vas. Sophias Ave, 11528 Athens, Greece

**Keywords:** Gastrointestinal bleeding, Hepatic artery, Aneurysm, Peripheral vascular disease

## Abstract

Endovascular management of small visceral artery aneurysms is an established treatment with satisfactory outcomes. However, when size exceeds 5 cm visceral aneurysms are considered as “giant” (giant visceral artery aneurysms or GVAAs) and management is significantly more complex. Between August 2007 and June 2019 eleven cases of GVAAs that were endovascularly treated were retrospectively reviewed and included in this single center study. Mean size was 80 mm (± 26.3 mm) x 46 mm (+ \-11.8 mm). Nine of the lesions were true aneurysms, and two were pseudoaneurysms. In 8 patients, the lesion was causing compression symptoms in the surrounding organs, one patient developed a contained rupture while 2 patients were completely asymptomatic. However, all patients were hemodynamically stable at the time of treatment. Technical success was defined as immediate complete exclusion of the aneurysmal sac, and clinical success as complete relief from clinical symptoms. Follow-up was performed with CT angiography, ultrasound and clinical examination. Mean follow-up was 45 months (range 6–84). Technical and clinical success were both 91%. Complications were one lack of control of contained rupture that was subsequently operated, one case of self-limiting non-target spleen embolization and one case of splenic abscess. Three patients died, one due to the contained rupture 15 days after procedure, the other two for other causes and occurred during the long-term follow-up. This series suggests that endovascular treatment of giant visceral artery aneurysms and pseudoaneuryms is a valid minimally invasive solution with very satisfactory immediate and long-term outcomes unless the aneurysm is already ruptured. A variety of endovascular tools may be required for successful treatment.

## Introduction

Visceral artery aneurysms (VAAs) are a rare and potentially life-threatening vascular disorder involving mainly the splenic artery but also the hepatic and the celiac arteries with incidence between 0.1% and 2% in the general population^1–3^. True aneurysms may occur as a result of atherosclerosis, fibro-muscular dysplasia, medial degeneration or arteritis and pseudoaneurysms due to inflammatory conditions, infectious diseases, and trauma^4,5^. Clinical presentation is nonspecific and diagnosis is often incidental. The main complication is rupture that would require emergency management as mortality may reach even 30%^6^. For true aneurysms, the risk of rupture is mainly related to the size of the lesion therefore VAAs are treated generally when their diameter exceeds 2 cm^7,8^. If their diameter exceeds 5 cm they are defined as “giant” (giant visceral artery aneurysms or GVAAs) and the risk of rupture is significantly increased^9–12^.

Treatment is nowadays endovascular as less invasive than open surgery^2^. Nonetheless, for the endovascular treatment of giant visceral aneurysms there are some technical challenges to be faced^11^. In this single center study, we report the outcomes of a series of GVAAs that were treated with a variety of tools from the endovascular armamentarium. .

## Materials and methods

We performed a retrospective review of the electronic database of our institution, looking for patients who underwent endovascular treatment of all VAAs in a 12-year period, (August 2007 to June 2019). Out of 124 patients, 11 patients with a GVAA were identified and enrolled in the study. The criterion to define a lesion as GVAA was one of the size measurements to exceed 5 cm in the pre-treatment Computed Tomography (CT) scan. CT analysis also provided information about the aneurysm anatomical features (diameter, neck length, distance from the aorta, diameter and number of the afferent and efferent arteries) as well as the relationship with the surrounding organs.

The endovascular treatment protocol that was followed was the same for all cases and included planning according to the information obtained from the CT scan in order to achieve sealing of the aneurysm. Preservation of the parent vessel was desirable when possible but given the large size of such lesions this was an extra challenge and in some cases decision to sacrifice the parent vessel was made given the presence of collateral circulation. Prior to the procedure, each patient was informed about the intervention and a written consent was obtained. Unless there was a rupture, a bolus dose of 2500 International Units (IU) of heparin was administered together with prophylactic antibiotics (1.2 g of co-amoxiclav). In case of covered stent treatment, a double antiplatelet regimen was established for 30 days (clopidogrel 75 mg/day and AAS 100 mg/day after procedure) and then mono-antiplatelet therapy with AAS 100 mg/day, indefinitely.

Technical success was defined as the immediate complete exclusion of the aneurysmal sac; clinical success was defined the complete relief from initial clinical symptoms. As ‘mid-term’ period was considered the first six months post treatment according to the commonly adopted terminology for aneurysm repair. All imaging was reviewed by two interventional radiologists with extensive experience in endovascular procedures (23 and 11 years).

Follow-up protocol consisted the combination of clinical examination and imaging and more specifically CT angiography at 1, 6, 12 and 24 months; at 36 months CT scanning was replaced by ultrasound imaging and continued annually.

## Results

Nine out of 11 lesions were true aneurysms of the splenic (5 cases) and the hepatic artery (4 cases), and two were pseudoaneurysms of the splenic and the left gastric artery, respectively. Two out of the 11 patients presented without any symptoms and the lesions were detected incidentally while in nine cases patients were presented with symptoms related to the presence of a GVAA and in particular abdominal discomfort and pain, lumbar pain, jaundice or even intermittent upper gastrointestinal bleeding (Table 1). Mean aneurysmal sizes at presentation were 80 mm (± 26.3 Standard Deviation (SD) x 46 mm (+ \-11.8 SD); the number of outflow vessels ranged from 1 to 4. Patient details and lesion features are described in Table 1. All patients, were treated within 24 h after the diagnosis. The patient that was presented with the contained rupture was treated few hours after detection.

**Table 1:** Patient’s characteristics.

Patient	Age	Sex (M/F)	True false aneurysm	Size (mm)	Symptoms /signs	Location (artery)	Access site	N° outflow vessels	Embolization techniques	Complications		Follow-up (months)
										Peri-procedural	Post-procedural	
1	68	M	T	88 × 140	None	Splenic	RF	4	Coils, Plug	None	None	48
2	74	M	T	63 × 55	None	Splenic	RF	3	Coils, Plug	None	Splenic abscess	72
3	27	M	T	52 × 70	Abdominal pain^a^	Splenic	RF	3	Coils, teflon wire pieces, Glue, Plug	None	Splenic infarction	72
4	69	M	T	100 × 40	Abdominal pain^a^	Hepatic	RF	4	Onyx, Teflon wire, Amplatzer Plug	None	None	48
5	49	M	T	56 × 30	Jaundice^d^	Hepatic	RF	2	Peripheral occlusion device (POD)	None	None	12
6	63	M	T	43 × 52	Back pain^b^	Hepatic	RF	1	Covered stent	None	None	84
7	65	M	T	95 × 36	Abdominal pain^a^	Splenic	LA	1	Covered stent (3)	None	None	12
8	36	F	F	59 × 33	Intermittent UGIB^d^	Left gastric	RF	2	Coils	None	None	18
9	69	M	T	130 × 35	Abdominal pain^a^	Hepatic	RF	4	Coils, POD, plug	None	None	6
10	73	M	F	60 × 42	Intermittent UGIB^d^	Splenic	RF	1	Covered stent (2)	None	None	24
11	71	M	T	55 × 45	Contained rupture	Splenic	RF	3	Coils, onyx, teflon wire	Acute rupture	Open surgery	15 days death

M: male, F: Female, T: true, F: False, RF: right femoral, LA: left axillary.

^a^Abdominal pain: patients presented with abdominal discomfort and pain, probably due to the large dimension of the aneurysm. After the endovascular embolization, a relieve of the pain was obtained in all patients.

^b^Back lumbar Pain: probably due to the compression of the celiac plexus. When patients presented with pain it was referred to the aneurysm after excluding other possible causes.

^c^Jaundice: due to the extrinsic compression of the main biliary duct with dilatation of the intrahepatic ducts. The patient was treated with a percutaneous biliary drainage and, subsequently, with an endovascular embolization obtaining a rapid decompression because of an aneurysm shrunk.

^d^Intermittent Upper Gastrointestinal Bleeding – UGIB: both patients had pseudo-aneurysmatic lesions presenting with moderate anemia resulting from slow bleeding arising from the gastric wall in contact with the pseudoaneurysm. The lesions were detected at the CTA, requested after endoscopic examination.

**Figure 1:**
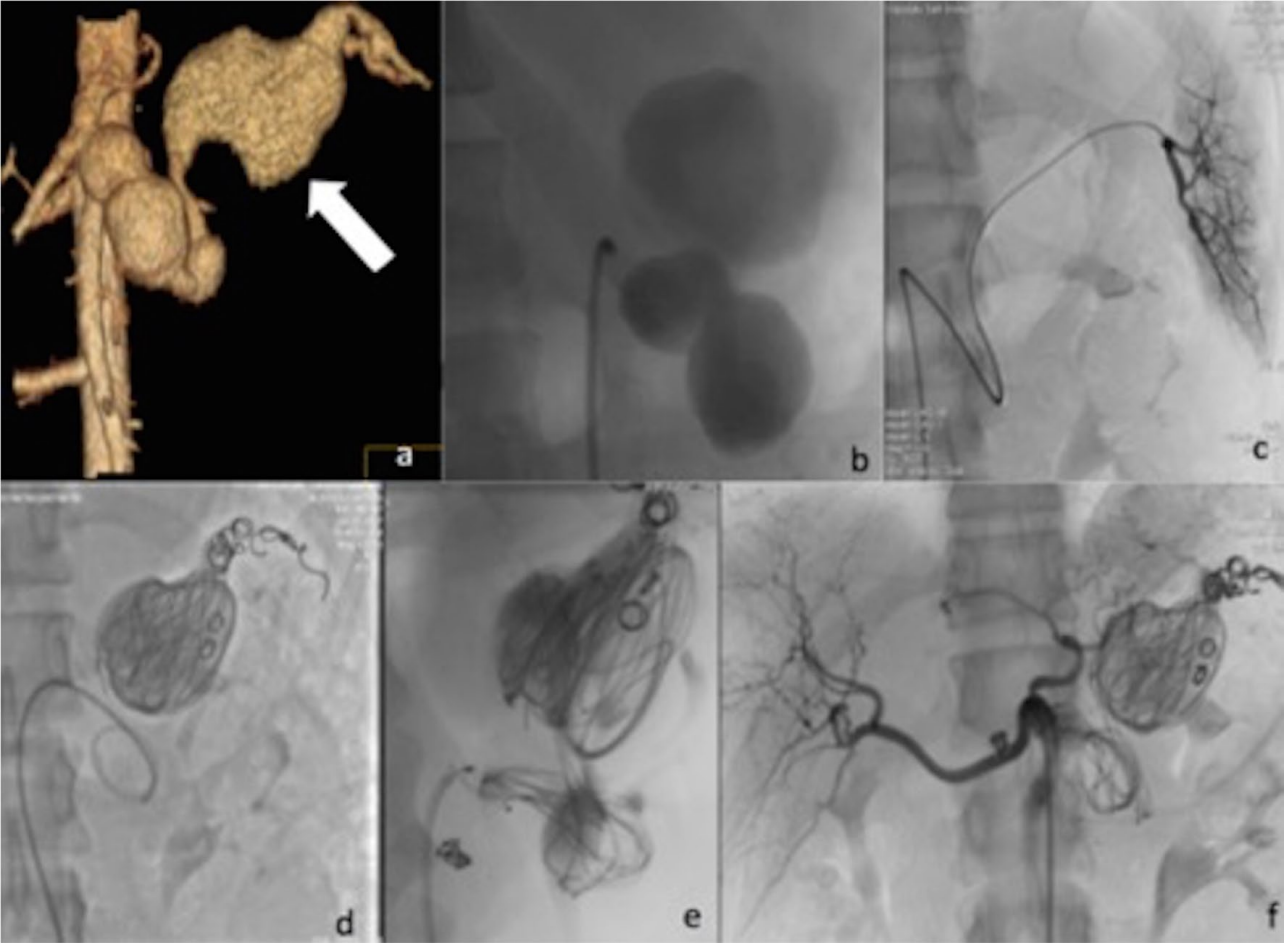
(**a**) Volume rendering contrast enhanced CT confirming the presence of multilobed fusiform splenic artery GVAA measuring 88 × 140 mm (arrow). (**b**) Angiogram confirmed the CT findings. (**c**) Selective catheterization of the distal splenic artery of “back door” of the aneurysm. (**d**,**e**) Endovascular exclusion was obtained by transcatheter “sandwich” embolization with Tornado coils (Cook Medical, Bloomington, Indiana, USA) and Jackson coils (Cook Medical, Bloomington, Indiana, USA) of the distal and proximal tract of the main artery and packing of the sac. Furthermore, inflow was totally stopped using a 8 mm detachable vascular plug (Amplatzer Plug, AGA, Plymouth, USA) in the proximal neck of the aneurysm. (**f**) Angiogram from the coeliac axis confirming satisfactory exclusion of the GAA.

**Figure 2:**
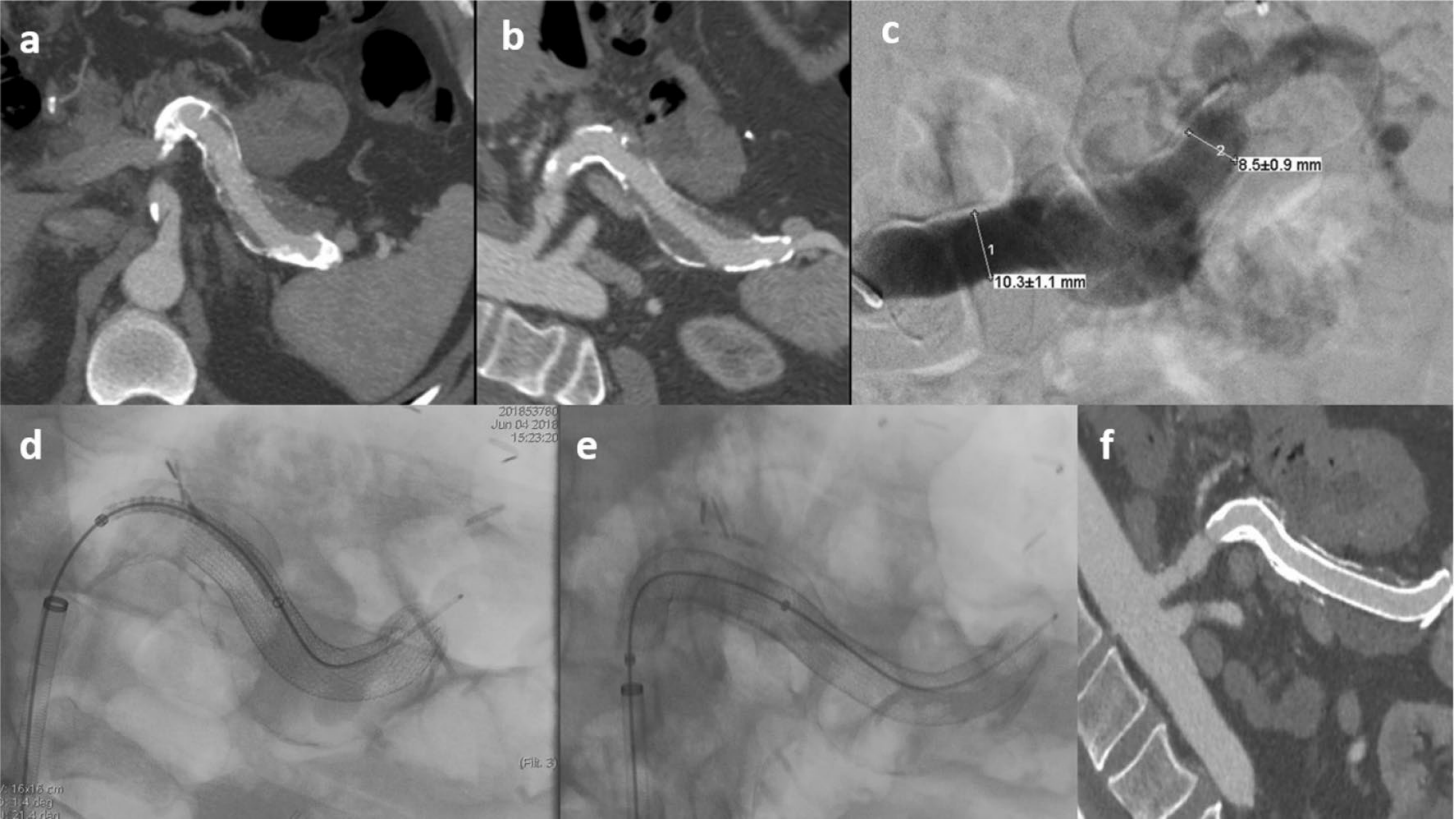
(**a**) CT scan in arterial phase demonstrated a 95 × 36 mm GAA of the splenic artery. (**b**) Curved reconstruction shows the complete extension of the aneurysm and the thrombosed part of the sack. (**c**) Angiogram confirming the aneurismal dilatation and allowing treatment planning. (**d**,**e**) Endovascular treatment was performed by positioning three overlapping covered stents (Viabahn; Gore, Falstaff, AZ) measuring in order from distal to proximal: 10 × 100 mm, 10 × 50 mm and 11 × 50 mm f) Angio-CT with curved reconstruction shows patency of the splenic artery with aneurysm complete exclusion.

Access was obtained via the right femoral access in all but one case where a left axillary access was retained as more suitable approach. Access sheath diameter ranged from 6 to 9 Fr. In eight cases the strategy that was adopted consisted in embolization of the distal feeding branch or “back door” and the proximal feeding vessel or “front door” of the lesion. Embolization of the aneurysmal sac was also performed in six of these cases, however not with the aim of complete embolic packing, as the size of the aneurysm was very large (Fig. 1). A large number and extensive variety of embolic materials (and their combination) were used for those eight cases. In particular pushable coils [Tornado and Jackson coils (Cook Medical, Bloomington, Indiana, USA), and Vortx (Boston Scientific, Natick, USA)], vascular plugs [Amplatzer Vascular Plugs (AGA, Plymouth, USA) and POD- Penumbra Occlusion Device (Penumbra, Inc., Alameda, California, USA), liquid embolics [Ethylene–Vinyl Alcohol Copolymer (Onyx LES, Covidien, Paris, France)—in a case that was also previously reported as case report^13^- and Nbutyl- cyanoacrylate (Glubran II, GEM, LU, Italy)] and – in the early days- even fragments from the Teflon-coating of an angiographic guidewire. In the remaining three cases, exclusion of the sac was obtained with the placement of one or more covered stents (Viabahn, W.L. Gore, Flagstaff, AZ, USA); an example is illustrated in Fig. 2.

The mean time of follow-up was 48 months (range 6–84 months). Technical and clinical success was obtained in 91% of the cases. Complete exclusion of the sac and complete relief from clinical signs or symptoms for the whole follow-up period was achieved in 100% of the cases.

Most cases were successfully treated in a single session. One case required a second treatment due to the difficulty in advancing a covered stent in the desired position; aneurysm exclusion was achieved the next day with front and back door embolization using multiple coils and a vascular plug.

Complications were divided in minor and major and were classified according to the Society of Interventional Radiology guidelines^14^. One minor complication occurred, consisting in asymptomatic ischemic changes of an area of the spleen; it was detected in the follow-up scan one month after the procedure. No change was detected in the subsequent follow-up scans and the patient remained completely asymptomatic.

Two major complications occurred. In one case the patient developed fever and malaise one-week post treatment. CT scan confirmed the presence of a 4 cm splenic abscess; it was successfully treated with antibiotics and no drainage or surgical intervention was required. No further abscess was detected in the follow-up scans. However, the complication was classified as major (class D) given the fact that the patient had to stay in the hospital.

The second major complication occurred to a 71-year-old man who arrived in the emergency department with a 55 × 45 mm distal splenic artery aneurysm associated with abdominal pain, low Hb level (5 g/dl) and a large perilesional and intraperitoneal hematoma; CTA excluded however active extravasation and the event was considered as a contained rupture. The patient- even though was not aware- also suffered from advanced cirrhosis with portal hypertension and thrombosis of the portal vein. Emergency endovascular treatment was agreed in a multidisciplinary setting and the patient was transferred immediately to the angio-suite. Treatment with sac and front and back door embolization was decided. However, during embolization of the sac and the three outflow vessels, with coils and Onyx, acute rupture of the sac occurred. The patient became hemodynamically unstable and an 8 mm balloon had to be inflated in the ruptured vessel to contain the rupture. The patient was immediately transferred to theatres for emergency laparotomy that was successful. However, he never fully recovered and died 15 days post procedure. The complication was classified as major (class F). Another two patients died at two and five years after the treatment, respectively, for causes not related to the aneurysm or procedure.

## Discussion

Aneurysms involving the visceral arteries represent an uncommon form of vascular disease that carries the risk of life-threatening haemorrhage if rupture occurs. Open surgery is the historical treatment but with a mortality rate even for elective cases described between 1.3 to 5% for true aneurysms and up to 9.4% for pseudoaneurysms^15,16^. For emergency cases, open surgery mortality may vary from 25 to 90%^15–18^.

Endovascular techniques, on the other hand, offer a minimally invasive alternative and provide benefits in terms of lower morbidity and higher survival for both elective and emergency setting with lower hospitalization time^1–8,19–22^.

There is no Level 1 evidence from prospective randomized comparison of the two techniques, however given the lower morbidity and the lower hospitalization rates visceral aneurysms are nearly exclusively treated by endovascular means^6^. In a recent systematic review by Kok et al.^23^, among 22 retrospective cohort studies with 643 endovascularly treated visceral aneurysms, technical success was 93.2% and distal visceral preservation rate was 99.3%. The rate of major complication was 3.5% and the thirty-day peri-procedural mortality was 1.5%, whereas, re-intervention rate was 4.6%.

However, scientific literature lacks in studies focusing exclusively in the management of GVAAs with only scattered reports^11^. Although there is no data about surgical management in this setting, it’s reasonable to assume that morbidity and mortality would increase proportionally for giant lesions. The long-term efficacy of GVAAs endovascular treatment has also been initially demonstrated in the retrospective study of Spiliopoulos et al.^24^ who reported a high clinical and technical success rate for true aneurysm with mean diameter of 49.4 ± 21 mm and for visceral pseudoaneurysms with a mean diameter of 25.1 ± 14.6 mm.

To our knowledge, our study represents the largest case series in literature focused on the endovascular treatment of such lesions. The anatomic distribution of GVAAs in our population included five giant splenic, four giant hepatic artery aneurysms and two giant pseudoaneurysms taking origin from the left gastric and the splenic artery, respectively. The endovascular treatment required a combination of several techniques and materials from the interventional armamentarium like coils, vascular plugs, liquid embolic agents and covered stents.

The “front door back door” technique is of paramount importance in order to diminish the risk of reperfusion; this requires embolizing all the efferent vessels. Selective catheterization of small arteries originating from a huge sac is challenging and in some cases partial embolization of the aneurysmal sac helps by reducing the flow and making more accessible the outflow vessels. In such cases, the use of liquid embolics may also be very useful as they may reach small efferent vessels^13^.

The use of covered stents, which would preserve patency of the parent vessel^20,25^, is not always technically possible, particularly when dealing with GVAAs, mainly due to the significant tortuosity of the parent vessel, due to the irregularity of the aneurysm or due to the size discrepancy of the proximal and distal landing areas. The use of some interesting techniques derived from the endovascular neurointervention experience has been applied in the case of GVAAs. For example, Gjoreski et al. reported a case of GVAA of the hepatic artery successfully treated with dual-layer stents placement as a flow-diverting option^26^. However, despite the advantage of these devices, such as the capability of preserving the parent vessels and the low profile, which allows an easier deployment, there are not any long-term information about their effectiveness in dealing with such big aneurysms, especially in terms of flow diverting power. Moreover, the long segments of the arteries involved in GVAAs, usually, still preclude their use.

In our experience stenting technique was used, successfully, in only three cases. In one case a covered stent was deployed from the celiac trunk to the splenic artery in order to exclude the entire aneurysmal common hepatic artery: this was possible due to the fact that the distal hepatic artery was dissected and blocked distally. In the other two cases covered stents were deployed to exclude splenic GVAAs.

Packing of the sac may also be performed using different embolic agents such as fragments of Teflon guide wires, long detachable coils that are nowadays available or even liquid agents in order to obtain a better thrombosis of the aneurysm and potentially decrease the risk of endoleak from small outflow vessels, not eventually detected during the procedure.

Technical and clinical failure occurred in only one patient that was the only one who presented with a contained rupture from a true splenic aneurysm. Partial end-organ infarcts (less than 50% of the splenic tissue) occurred in two patients, and one of them developed an abscess that was resolved after antibiotic therapy without the need of drainage. Clinical and imaging follow-up demonstrated the patient good status and the satisfactory thrombosis of the aneurysms with no evidence of sac growth or endoleaks.

This study’s limitations include the retrospective and single center design and the small number of patients. However due to the nature and the rarity of this specific pathology it is rather challenging to design a prospective study and numbers will inevitably be low.

In summary, this single center study assessed the immediate and mid-term safety and efficacy of the endovascular treatment for visceral aneurysms that are larger than 5 cm with good outcomes. Treatment of such lesions might require the combination of endovascular tools and may be linked to an increased procedural cost, however it is still less invasive than open surgery and the satisfactory mid- and long-term outcomes suggest that it should be considered as the first line treatment when the necessary expertise and resources are available. Patient’s hemodynamic condition would also play a crucial role in triage and outcomes and need to be considered carefully prior to treatment.

### Ethics statement

Due to the retrospective nature of the study, informed consent was waived by the ethics committee (University La Sapienza of Rome committee). All methods or experimental protocols were approved by the local Institutional review board (Sant’Andrea Hospital) and were carried out in accordance with relevant guidelines and regulations.

## Abbreviations

GVAAs: Giant visceral artery aneurysms and pseudoaneurysms
VAAs: Visceral artery aneurysms
CTA: Computed tomography angiography
VR: Volume rendering
IU: International units
